# A Practical Guide to Pilot Testing Community-Based Vaccination Coverage Surveys

**DOI:** 10.3390/vaccines11121773

**Published:** 2023-11-28

**Authors:** Dale A. Rhoda, Felicity T. Cutts, Mary Agócs, Jennifer Brustrom, Mary Kay Trimner, Caitlin B. Clary, Kathleen Clark, David Koffi, Jean Claude Manibaruta, Alieu Sowe, Rajni Gunnala, Ikechukwu U. Ogbuanu, Marta Gacic-Dobo, M. Carolina Danovaro-Holliday

**Affiliations:** 1 Biostat Global Consulting, 330 Blandford Drive, Worthington, OH 43085, USA; 2 Department of Infectious Disease Epidemiology, London School of Hygiene and Tropical Medicine, Keppel Street, London WC1E 7HT, UK; 3 American Red Cross, 431 18th Street NW, Washington, DC 20006, USA; 4 Cabinet d’Appui au Développement Sanitaire, Abidjan, Côte d’Ivoire; 5 Burundi Country Office, World Health Organization, Boulevard de I’Uprona-Rohero II, Bujumbura P.O. Box 1450, Burundi; 6 Ministry of Health and Social Welfare, The Quadrangle, Banjul, The Gambia; 7 US Indian Health Services Area Office, Indian Health Service, 40 N Central Ave #600, Phoenix, AZ 85004, USA; 8 Child Health and Mortality Prevention Surveillance (CHAMPS) Network, Crown Agents in Sierra Leone, 28 Bathurst Street, Freetown, Sierra Leone; 9 Department of Immunization, Vaccines and Biologicals, World Health Organization, Avenue Appia 20, 1211 Geneva, Switzerland

**Keywords:** vaccination coverage, data collection, pilot test, household survey, vaccination, immunization, surveys, vaccine-preventable diseases

## Abstract

Pilot testing is crucial when preparing any community-based vaccination coverage survey. In this paper, we use the term *pilot test* to mean informative work conducted before a survey protocol has been finalized for the purpose of guiding decisions about how the work will be conducted. We summarize findings from seven pilot tests and provide practical guidance for piloting similar studies. We selected these particular pilots because they are excellent models of preliminary efforts that informed the refinement of data collection protocols and instruments. We recommend survey coordinators devote time and budget to identify aspects of the protocol where testing could mitigate project risk and ensure timely assessment yields, credible estimates of vaccination coverage and related indicators. We list specific items that may benefit from pilot work and provide guidance on how to prioritize what to pilot test when resources are limited.

## 1. Introduction

Community-based vaccination coverage assessments are a unique type of household data collection involving the coordination of numerous logistical, administrative, and data management factors [1]. Assessments of this kind are often surveys that employ a rigorous sampling protocol to estimate coverage for a subnational or national population. In other situations, a targeted set of households in a more limited geographical area are interviewed, and the resulting data are used to inform local or regional vaccination program management. In this paper, we employ the word *survey* informally to mean either sort of project. Vaccination coverage interviews involve recording subjects’ (usually children’s) personal vaccination history from home-based records (HBRs), from caregiver recollection using probing [2], and sometimes from health facility-based records (FBRs). 

Pilot testing can help survey coordinators strike a favorable balance among pressures on the project budget, timeline, and quality of implementation, analysis and reporting [2] Survey practitioners use the term pilot test in a variety of ways—ranging from early small-scale tests of a specific data collection feature to a full test of the entire study protocol [3,4]. In this paper, we use the term pilot test to mean informative work carried out before the survey protocol is finalized for the purpose of guiding decisions about how the work will be conducted. Pilot testing has the potential to benefit all stages of the work. We organize lessons learned and recommendations around four areas:

- Sampling
- Survey instruments
- Field procedures
- Data management, data quality, and data analysis

This paper provides practical guidance for informing decisions regarding each project stage. We first present examples of pilot findings we used to refine survey protocols and instrumentation for childhood vaccination coverage assessments. After describing the pilot test examples, we present general recommendations followed by a thorough list of survey elements that might benefit from pilot work. We then conclude with a framework for prioritizing what to pilot when resources are limited. Although there is likely benefit to piloting potential methods of disseminating results to stimulate action, that topic is beyond the scope of this paper.

## 2. Illustrative Examples of Vaccination Coverage Pilot Tests

This section describes illustrative vaccination coverage data collection pilot efforts and corresponding lessons learned. We selected these seven pilots because they are excellent models of preliminary efforts that informed the refinement of data collection protocols and instrument(s). All pilot procedures described in this paper are in line with the current World Health Organization’s (WHO) guidance on vaccination coverage surveys [2,5]. An ethical review for each survey was carried out in accordance with the requirements from each country. Table 1 lists the specific activities, components explored during pilot testing, and findings that informed future work. 

### 2.1. American Red Cross (AmCross) 5-Point Plan Rural Pilot

The 5-Point Plan (5PP) is a methodology developed by AmCross to identify pockets of zero-dose and under-vaccinated children and understand the reasons they are under-served [6]. In November 2019, AmCross, the Kenya Red Cross Society (KRCS), the Kenyan Expanded Program on Immunization (EPI) team, and the Ministry of Health (MoH) piloted the 5PP in Bobasi, a rural sub-county in Western Kenya with the intention of scaling up the 5PP program in Kenya and elsewhere. A total of 293 Red Cross volunteers working in 29 teams aimed to conduct face-to-face interviews with every household in the sub-county. The teams visited over 60,000 households in a week-long period. The pilot identified several pockets of children without cards or HBRs or who were missing one or more age-appropriate vaccinations. To learn why these children were un- or under-vaccinated, a small team conducted focus group discussions and one-on-one interviews with the children’s caregivers, which were complemented with interviews of frontline healthcare workers who provide vaccination services in Bobasi. 

During the 5PP rural pilot, the project team evaluated several logistical and administrative procedures and project strategies, including adequacy of community awareness, project team training, the household questionnaire, and data management and storage procedures (Table 1). Following the pilot test, written and verbal feedback was obtained from supervisors, independent monitors, AmCross staff and consultants, KRCS, and the MoH. Project leaders documented lessons learned, including a need to standardize volunteer training materials and pedagogy; translate the Open Data Kit (ODK) household questionnaire into the local language; refine, add, and remove selected questions; upgrade equipment and procedures to avoid data collection errors and equipment malfunctions (e.g., phone crashes); address deficits in data quality; and develop a standardized process to revisit a select subset of the survey households (Table 1).

### 2.2. AmCross 5-Point Plan Urban Test

In November 2021, 5PP project staff piloted a protocol for dense urban settings with informal settlements and high-rise buildings in the Pipeline neighborhood of Nairobi, Kenya. Over three days, two teams of Red Cross data collection volunteers visited 2791 households. Items of interest included data quality, volunteer training, revisits, and challenges of navigating in a densely populated urban context. One of the priorities that stemmed from the earlier Bobasi pilot with respect to data quality was to develop the capacity to validate the accuracy of the date of birth and vaccination status information recorded by interviewers. In this later urban exercise, interviewers took digital photos of each available HBR. Project staff, who were different from the interviewers and knowledgeable about vaccination, later reviewed every photo and scored the accuracy of the data entered. Staff also assessed the feasibility of navigating and conducting interviews in poorly lit, high-rise buildings, including interviewers’ ability to take clear photographs of HBRs and record the data needed to facilitate revisiting households where no eligible respondent was at home during the initial visit. Standardized volunteer training was also tested during this pilot in response to one of the main findings from the earlier Bobasi pilot.

Project leaders convened a workshop of representatives from KRCS and the MoH, Red Cross volunteers, and AmCross staff in early November 2021 to collect lessons learned from the urban pilot. Participants first completed an anonymous assessment of their opinions related to their experience. Attendees next split into small discussion groups and responded to three questions: *What went well? What did not go well? What could be carried out differently in the future?* Workshop leaders summarized this feedback thematically in a final report [7]. Findings from the workshop that were used to improve future implementation efforts included identifying the need to amend the ODK form to tag non-residential locations (e.g., stores) or vacant units within multi-family dwellings (e.g., high-rise buildings); encouraging teams to rely more on landmarks and line list information for revisits; expanding the volunteer training to provide more information about revisits; and a need for trusted community escorts to address safety and security concerns (Table 1).

### 2.3. Bangladesh Survey Pilot

In 2014, the team responsible for updating the WHO’s Vaccination Coverage Cluster Surveys Reference Manual [2] conducted pilot work in Bangladesh to shed light on recommendations being considered for the manual. The team worked in the district of Bogra, northwest of Dhaka on the West bank of the Jamuna River (Bramaputra River), in five rural clusters in the Upazila of Sariakandi and five semi-urban clusters in the town of Bogra. The work examined two cohorts, children aged 0–11 months and 12–23 months, and used an adapted version of the latest EPI national survey questionnaire [8]. The pilot study explored a variety of dimensions, including testing household selection and sampling methods; concordance of HBR and caregiver recall data; feasibility of visiting health facilities to collect vaccination data; use of electronic data forms to capture vaccination evidence; and operational measures, including study-related time, cost, and logistics (Table 1). Findings from the pilot used to refine subsequent study protocols and instruments included identifying a need for additional supervision for field teams, determination that FBR data did not provide much additional vaccination evidence above HBRs or caregiver recall in this setting, and a need for a long lead time when requesting copies of official maps from government offices (Table 1).

### 2.4. Gambia Operational Study 

In September 2022, a team affiliated with the Gambia MoH and Gambia Bureau of Statistics conducted an operational study to test field procedures in preparation for an upcoming routine vaccination coverage survey. Study staff completed a three-day training covering the project’s purpose, tools, maps, and field testing, followed by 10 days of data collection and a 14-day transcription period. The focus of the data collection was evidence of routine vaccination (using HBR and recall) among children aged 12–35 months in 19 urban and 13 rural clusters. The pilot study assessed the agreement between HBRs and caregiver recall for 20 vaccines, coverage differences between HBR and recall, differences in transcription time for data entered in the field versus in an office setting, and barriers and enablers to transcription. Lessons learned from the pilot included the need for good planning, thorough testing and re-testing of electronic data collection tools, and the importance of high-quality training and supervision. On average, transcription of information from photographed HBRs conducted in an office setting took roughly half as much time as transcription conducted from the original HBRs in the field (Table 1). 

### 2.5. Mali Survey Pilot

In preparation for the second phase of a routine vaccination coverage survey in Mali, a team from Appui au Développement Sanitaire (ADS) Côte d’Ivoire and L’ Institut National de la Statistique (INSTAT) Mali conducted a pilot study to address issues known to be challenges in previous vaccination coverage surveys. The first phase, following the pilot described here, took place from June to September 2022 and covered six districts of Bamako (Capital) and three districts of the Koulikoro Region. This first phase surveyed 9570 children aged 12 to 35 months.

The objectives of the pilot included cataloging all types of vaccination documentation in circulation to ensure staff training materials included photographs of each type of documentation and instructions tailored to each. As part of the pilot, the team tested a two-level process for cross-checking data entry accuracy and a training protocol designed to provide field staff with experience interpreting challenging vaccination cards. The pilot also explored the correspondence between vaccination status assessed via caregiver recall compared to health FBRs. During the pilot phase, the team encountered up to 20 different types of vaccination documentation—a big difference from the one official vaccination card or HBR—much of which was outdated. Staff responsible for cross-checking data entry accuracy noted errors in the transcription of vaccination information from notebooks to the study database. Lessons learned from the Mali pilot that investigators will use to inform future vaccination coverage surveys include the following: catalog and train on all variations of HBRs that interviewers are likely to encounter; train interviewers to look for improvised entries when older cards do not list newer vaccine doses; double-and triple-check the date of birth and dates of vaccination; and use colloquial names for vaccines. 

### 2.6. Measles and Rubella Vaccination Campaign Evaluation Pilot Study, Burundi

In 2022, the Institute of Statistics and Economic Studies of Burundi (ISTEEBU), in collaboration with the Ministry of Public Health and the Fight against AIDS and its technical and financial partners, evaluated an approach for conducting a post-measles and rubella vaccination campaign coverage survey. The primary objective of the pilot was to assess the feasibility of visiting health facilities to obtain vaccination records or FBRs for children whose caregivers said they were vaccinated but were lacking an HBR. 

Study staff participated in three days of training and a four-day data collection period. One phase of the pilot involved testing the methods and data collection tools planned for use in the main survey. Five field teams collected data in households and health facilities in ten enumeration areas of five provinces (Bujumbura Mairie, Cibitoke, Kirundo, Makamba and Ruyigi). Teams subsequently participated in a plenary session in which they provided a status report on the data collection for their assigned area, the difficulties they encountered, and corresponding solutions. Study leaders used information from the plenary session to make improvements to the questionnaires and computer applications that will be used in the upcoming main survey. Helpful lessons included increasing the number of staff available for listing households in large clusters. For children without cards whose records were found in the health facility, pilot findings indicate the value of transcribing the FBR dose dates onto a card, photographing the card, and presenting the card to the caregiver when feasible (Table 1).

### 2.7. National Stop Transmission of Polio (NSTOP) Pilot, Nigeria 

The NSTOP program was established in 2012 to accelerate polio eradication efforts in Nigeria. The program places staff at national, state, and local government area (LGA; equivalent to district) levels to strengthen routine vaccination service delivery with the aim of eradicating polio [9]. In preparation for conducting routine vaccination coverage data collection in 40 polio high-risk LGAs across eight states in Northern Nigeria, the field team conducted household visits to pilot test the survey instrument and data collection procedures, including locating assigned clusters using global positioning system (GPS) navigation. Leaders from NSTOP and the U.S. Centers for Disease Control and Prevention first trained senior and team supervisors during a three-day period in Abuja and conducted a pre-pilot (field testing) of the data tools in an Abuja suburb. Following initial minor modifications to the field guides and data collection instruments, the master trainers then conducted a two-day interviewer training in one of the eight states (Kaduna State) as part of the survey pilot. During the pilot phase, a surplus of interviewers was recruited and trained. Only interviewers who performed well during the training and post-training evaluation were retained for the primary data collection effort. The pilot test took place in two Kaduna LGAs with varied characteristics (urban/rural, geographic/population size, administrative vaccination coverage estimates, etc.). Lessons learned from the pilot used to improve the data collection protocol included strengthening the supervisory structure of data collection teams; reducing the number of clusters per day each interview team was expected to cover; leveraging the utility of GPS navigation to help survey teams locate assigned clusters; and deploying a remote monitoring system using GPS information and satellite imagery to confirm household structures remotely due to security issues which prohibited command center staff travel out of Abuja (Table 1) [9].

## 3. Recommendations

This section begins with some general recommendations, followed by a thorough list of survey elements that could benefit from pilot work, and concludes with considerations for how to prioritize which elements to pilot when time and resources are limited.

### 3.1. General Recommendations

The following list of general recommendations was informed by all seven of the illustrative case studies featured in Section 2. These recommendations are applicable to any pilot or full-scale household survey project.

**Enlist assistance from analysts with local vaccination expertise**. For vaccination coverage survey and vaccination modules in multi-indicator surveys, we recommend advanced analyses be conducted by specialists in the field of vaccination to ensure analyses account for elements such as rapidly evolving vaccination schedules, variability in vaccination cards or HBRs and idiosyncrasies in how they are completed (e.g., writing in pencil the next scheduled visit date versus writing in pen the dates of vaccination doses), and common terminology used in a country to refer to vaccines and vaccine-preventable diseases [10].

**Solicit feedback on earlier data collection efforts from vaccination stakeholders.** Teams should meet with key stakeholders to ask about the credibility of similar surveys conducted in the same country: *Were the goals achieved? Were the results believable? Controversial? Why? Did the survey influence important decisions and how is that influence regarded now with the benefit of hindsight?*

**Learn from survey teams with recent in-country experience.** Teams will benefit from meeting with others who faced similar challenges or used the same resources they will use (e.g., same sampling frame, primary sampling unit [PSU] maps, data collection hardware and software, logistics and data management teams). Helpful insight may be gleaned even if the topic of the earlier team’s work was not vaccination coverage. Ask: *What went well? What went poorly? What do they wish they had known at the start?* If appropriate, consider asking teams with prior experience to provide constructive feedback on the new project’s plans.

**Identify key decisions that need insight from pilot work.** Survey teams will want to synthesize feedback on earlier work with a short list of their project’s goals and make a list of possible pilot study topics and questions such as the following: *What are earlier weaknesses and pitfalls you want to avoid? What protocol elements could be realistically added to improve the credibility and utility of the study’s outcomes? What could be removed or simplified without incurring risk or compromising quality? Which of the proposed tools are unfamiliar and untested?*

Scope pilot testing effort appropriately. To be as informative as possible, a pilot might follow the 5PP model and test all procedures before the full survey begins [11]. Trying to pilot in as many different settings as possible (and especially dense urban versus sparse rural settings) can be particularly relevant: different settings present varying challenges that may require the field teams to depart from the survey protocol. 

**Start early**. Ideally, the project team should conduct pilot testing in advance of the primary data collection effort with sufficient time to refine survey instruments and protocol elements and provide retraining if needed [2] and allow time to obtain (and train on) better equipment if needed. When the pilot is a small token effort or an afterthought, there is little opportunity for the team to adjust the survey design or tools.

**Engage community leaders**. If the pilot includes substantial fieldwork, it will be helpful to engage community leaders early in the survey planning process. This is important for security and for improving the overall cooperation of the community to be willing participants. It is also critical to provide leaders with sufficient opportunity to ask questions.

**Allocate sufficient time and money to pilot testing.** Piloting key aspects of the protocol is an investment in the credibility of the survey results and the project organizers; thus, it often warrants a notable place in the project’s schedule and budget. 

**Document pilot findings**. Vaccination coverage data collection teams should document findings in final survey reports and strongly consider publishing findings based on their pilot testing experience to help other teams who plan similar work.

### 3.2. Survey Elements to Pilot Test

In contrast to the general recommendations, which are largely applicable across projects, determining which specific survey elements would benefit most from pilot testing is a more nuanced process. The tables in this next section list elements for teams to consider pilot testing, organized by project stage:

- Table 2 presents options for pilot testing sampling procedures, which can be critical for ensuring that the survey sample is representative.
- Table 3 lists options for pilot testing survey instruments, which will increase the likelihood that respondents will interpret the questions as intended and that interviewers will capture responses correctly.
- Table 4 includes a list of field procedures for pilot testing, which can maximize the likelihood that data collection processes will be carried out efficiently.
- Table 5 provides data management, data quality, and data analysis components to consider for pilot testing so data will be captured, stored, and interpreted accurately.

### 3.3. How to Prioritize Survey Elements to Pilot Test When Resources Are Limited

Every survey occurs in a very specific context and is carried out by a team that has unique strengths and weaknesses. Our seven examples represent a variety of real-world situations, but do not, of course, span all possible contexts. Each survey steering committee will need to assess their team’s capabilities, survey-related goals, and resources, to discern which items from Table 2, Table 3, Table 4 and Table 5 are most relevant and warrant priority consideration for pilot study investigation. When it is not practical to pilot every process in the protocol, we suggest that high-priority candidates include (a) design choices that could substantially increase data quality but also increase the budget or lengthen the survey implementation timeline, (b) protocol components that are untested or new to the project team, and (c) hardware and protocols for electronic data collection. 

Two high-cost/(possibly) high-reward field procedure decisions are whether to collect data from FBRs and how to collect and curate photographs of HBRs. In countries with low HBR availability and facilities that keep vaccination records well organized, stakeholders’ confidence in coverage outcomes and the base of data for timeliness outcomes may be substantially boosted by visiting health facilities and matching FBRs with records from household interviews. To be carried out successfully, this process requires considerable organizational liaising and attention to technical detail. It also requires understanding where to look for records of children who received vaccination services at multiple facilities. Research teams should spend adequate time during the pilot visiting health facilities and learning how to optimize the yield of good data if they plan to add this component to their survey design. In some contexts, FBRs are organized by workday rather than by recipient child, and it would be nearly impossible to have the longitudinal vaccination story of an individual match records with those from household interviews. In that situation, the FBR component should be dropped from the data collection protocol. 

Well-curated clear photographs of HBRs can improve data quality without needing to revisit households to correct mistakes. However, including photographs in the protocol requires data management workflows and a sufficient budget to have humans review those photos with careful attention to detail. Photos will require extra storage capacity on data collection devices, extra bandwidth for uploading to the server, the matching of often multiple photographs to an individual child, and extra procedures to compare the dates entered in households with what is seen on the images. One novel idea, illustrated in the Gambia example described above, is to have interviewers collect data from caregiver recall and collect excellent HBR photos. In lieu of asking field-based staff to enter HBR dates into touchscreen devices while visiting respondents’ homes, office-based staff subsequently examine the HBR photos in an office setting—possibly at a multi-screen workstation—and enter dates there using either a touchscreen device or a keyboard. This approach can reduce survey costs, save time in the field, and mitigate data entry errors from touchscreens, but it requires careful attention to obtaining high-quality photos while in the respondent’s home [23]. 

If one of the data collection objectives is to characterize vaccination timeliness or the prevalence of missed opportunities for simultaneous vaccination, the research team needs a plan to minimize date data entry errors and check dates that seem illogical or impossible to ensure the data collected are of high quality. These objectives depend on accurately recording each child’s date of birth and date of each vaccine dose. If the protocol includes using a calendar of local events to narrow down the interval that includes the child’s birthdate, data collectors should practice this in a pilot to understand whether it is well understood by caregivers. Teams should validate the responses, when possible, by comparing dates of birth from caregiver recollections with those on HBRs or FBRs or other official documents.

Any tools or elements of the proposed data collection workflow that are new to the team should be prioritized for testing. Complex data collection protocols and measures that are central to survey goals will warrant thorough testing. Research teams will benefit from contacting other teams who have used the same tools and should try the tools in realistic settings early enough to adapt them or drop them in favor of alternatives if they do not work.

Electronic devices for data capture have become the default standard in recent years even in low-income countries. Although tremendous progress has been made in simplifying many aspects of using touchscreen phones and tablets, we noticed that a recurring theme in our conversations was problems with hardware, software, and connectivity and a post hoc sense that those problems could have been mitigated or eliminated if we had conducted additional rounds of testing before going to the field. Our collective advice is to hire key project staff who are hardware and software savvy; test all the processes that involve the devices in realistic settings; and re-test after each change in the software and each change in the electronic questionnaire.

Finally, if there are team members who are new to vaccination coverage surveys or new in their roles, then extra investment in training and testing is warranted. Some data collectors may need to be dropped or assigned other duties if they do not show promise. If training is brief with little time in the field, or if training is diluted through a cascade or train-the-trainer model, experience suggests that data collected for the first week or more may be of very poor quality because staff are still learning their roles. We consider practical training to be a separate issue from piloting, but it is important enough to mention here.

### 3.4. Limitations and Additional Considerations

Key considerations for implementation of any pilot test are the time and monetary costs of the pilot relative to the knowledge likely to be gained. Unfortunately, we are not able to comment on this issue because none of our featured case studies explored time or cost factors systematically.

## 4. Conclusions

Pilot testing aspects of a vaccination coverage survey protocol is a prudent investment in the success, credibility, and eventual influence of the survey [4,51,52,53,54,55]. Lu Ann Aday put it well:

*No survey should ever go into the field without a trial run of the questionnaire and data collection procedures to be used in the final study. One can be sure that something will go wrong if there is not adequate testing of the procedures in advance of doing the survey. Even when such testing is done, situations can arise that were not anticipated in the original design of the study. The point with testing the procedures in advance is to anticipate and eliminate as many of these problems as possible and, above all, to avert major disasters in the field once the study is launched [15].*

We echo these sentiments and add that piloting is helpful for deciding which high-cost protocol elements are likely to add valuable insights to the survey. We recommend that stakeholders devote time and budget in the early stages of planning to use pilot work to inform decisions about the survey protocol. Further, project leaders should foster a team-wide expectation that the project plans will likely be revised because of pilot work and that some things may need to be piloted more than once. Ideally, project leaders should share their experiences and insights to benefit downstream teams who may carry out similar work.

## Figures and Tables

**Table 1:** Illustrative pilot studies.

Pilot Component	Findings from Pilot that Informed or Will Inform Future Work
**Example 1: AmCross 5-Point Plan (5PP) Rural Pilot (November 2019)**	
Community awareness of project	The steps taken to engage the Ministry of Health (MoH) and local partners were useful and critical. Project leaders identified two main areas for improvement: engaging with community leaders as early as possible and communicating clearly about project dates.
Recruitment procedures and staffing	Project leaders were generally pleased with the recruited supervisors and Red Cross data collection volunteers. The organizational chart was updated to include additional staff, including a deputy logistics officer and a focal person for the data collection operations center.
Training procedures	Team supervisors were trained first; each supervisor then trained the volunteers on their team. This led to variability in the content and thoroughness of training. In subsequent work, volunteer training was standardized, expanded from a half day to a full day, and delivered by supervisors in the local language supported by 5PP implementors. Training materials were updated to clearly cover aspects of the fieldwork that some staff struggled with, including the use of project technology.
Volunteer compliance with safety protocols	Not all volunteers wore Red Cross-branded clothing and some experienced threats to their safety during fieldwork. Project leaders decided to make Red Cross clothing mandatory for work and supply volunteers with whistles to use as an alarm.
Household questionnaire	Some volunteers had difficulty using the English language questionnaire. Translation of the household questionnaire into the local language(s) was incorporated as a standard step for future work. Some questions were revised for clarity. For example, a question collecting a household’s address was amended to be more useful in contexts where dwellings do not have a street address.
Communication protocols	Supervisors and operations center staff requested more information for monitoring data collection progress. This led project leaders to develop additional daily monitoring reports and add steps to send daily updates and reports to supervisors.
Equipment performance	On the first day in the field, many of the mobile phones used to collect data crashed and had to be reset. A new model of phone was used in subsequent work, and stress tests and steps to configure and update phones before fieldwork were added to standard operating procedures.
Data management and storage processes	Inefficiencies in data upload and download procedures were identified during the pilot. Mobile phones used to collect data did not have connectivity; therefore, volunteers met up with their team at midday and at the end of the day to upload data from a WiFi hotspot. The midday team meeting required a pause in the work and extra travel time; consequently, this step was dropped for future work. To process the day’s data and generate reports, operations center staff first had to manually download a data file. Automatic data downloads were implemented following the pilot.
Data quality and reliability	Several discrepancies in information collected in original interviews and follow-up interviews led to concerns about data quality. These concerns motivated updates to the household questionnaire and to volunteer training. Questionnaire updates included requiring the double entry of key fields, such as date of birth, and adding steps to take photos of vaccination records for thorough review in a subsequent pilot. Training materials for the data collection volunteers were revised to emphasize careful data entry and allow more time to practice using the questionnaire.
Mapping and reaching households and missed areas for revisit	Finding target households for revisit interviews was difficult in many cases. The household questionnaire was revised to collect the head of household name to facilitate identification of households and revisit line lists were updated to include head of household name and the nearest landmark.
**Example 2: AmCross 5-Point Plan Urban Test (November 2021)**	
Data quality and reliability	This pilot assessed the adequacy of measures taken to improve data quality following the 2019 rural pilot, namely updates to the household questionnaire and to volunteer training. After data collection, program staff reviewed photos of 219 HBRs and scored whether date of birth and vaccination records were entered correctly. The data entry error rate was very low, which increased confidence in the measures implemented to improve data quality.
Training procedures	The improved data quality and the performance of Red Cross volunteers during fieldwork illustrated that the standardized volunteer training was successful and should continue. Particularly on the first day of work, some volunteers had difficulty using the phone-based questionnaire, indicating a need for additional hands-on practice during volunteer training.
Household questionnaire	Following this pilot, the questionnaire was modified to include a process for noting (and ignoring) non-residential and vacant units inside high-rises and other multi-family dwellings. The questionnaire’s steps for photographing a vaccination record were developed based on the layout of a standard HBR in Kenya, but this pilot revealed that to capture the relevant information from a non-standard record, an additional photograph was sometimes required. The questionnaire was updated to allow additional photos to be taken.
Equipment performance	Mobile phone crashes were not an issue during this pilot, following the adoption of a new phone model and implementation of standard procedures to test, update, and configure phones before fieldwork.
Field navigation	Teams had difficulty using maps to move through the study area and find households for revisits. Volunteer training was subsequently updated to rely more on landmarks and line list information and include more general information about revisits. Identifying high-rise buildings for revisit was difficult using the information available on line lists; thus, the Open Data Kit (ODK) form was revised to collect more information that could be used to find a particular building, including how neighborhood residents refer to the building and names of nearby landmarks.
Safety protocols	The pilot revealed the need for trusted community escorts to accompany data collection staff in some locations.
**Example 3: Bangladesh Survey Pilot (2014)**	
Protocol adherence	After training, field staff were still concerningly slow and exhibited confusion about survey methods and their rationale. After several days of real data collection with extra supervision, staff became acceptably proficient.
Value of collecting FBR data	Pilot work explored whether FBRs would provide documented records for a notable portion of children who would otherwise lack them and whether the FBR evidence would be consistent with (update or overrule) caregiver recall to a degree that justified investment in obtaining and curating FBR data. HBR availability was high, and the FBR data did not provide much evidence that was lacking from HBRs or from caregiver recall. If the pilot area was representative, then collecting data from FBRs in that region of Bangladesh would not yield notable improvements to the coverage outcomes.
Availability, accuracy, and usability of maps for identifying sampling area boundaries	There were long delays in obtaining official government maps. The ad hoc maps that were created with assistance from local experts showed only partial agreement with the government maps that were eventually delivered. Consequently, the data collection team learned they needed to begin liaising with map-holding authorities early in the planning process.
HBR and FBR photo usability	The fieldwork showed the need to review photo quality immediately and take additional photos if there was glare or blur. Matching children from households with their records in the health facility was extremely labor intensive, which underscored the need to allocate sufficient resources to this task. Facility-based register pages were large and some of the writing was small and cramped, emphasizing the need to use high-resolution photographs with adequate resolution to zoom in on fields of interest later.
Data entry errors using touchscreen devices	Upon comparison of questionnaire data with review of HBR and FBR photos, some data entry errors were observed, especially errors in dates. This suggested a need to take measures to either engage in double entry with concordance checking or plan enough resources to review all suspicious dates from photos and review a random portion of non-suspicious dates from each data collector.
Worker training	The time-and-motion portion of this study was compromised because the week-long training period for the data collection teams was not adequate. The first week of data collection was inefficient because many teams were still learning their jobs. The timing of work in this early period was therefore not comparable with the timing of work later in the pilot. Persons assigned to measure task-related timing had to be reassigned to give basic supervision and instruction on how to do the work. Some workers never became effective at the job, suggesting that organizers should enroll enough workers in training so they could afford to dismiss those who do not pass a competency exam at the end of the training period.
**Example 4: Gambia Operational Study (September 2022)**	
Transcription efficiency	Transcription was much more efficient when the task of transferring information from photographs of HBRs to the survey database was performed in an office setting with a computer and keyboard (~3–4 min per card), and the option to project and enlarge the picture from a different device or screen was available, compared to transcription in the field using a touchscreen tablet (~6–7 min per card).
Data collection protocol and standard operating procedures	Good planning is key. The operational study underscored the importance of ensuring clear division of responsibilities of data collectors working within the same cluster. The team also learned the importance of ensuring that each respondent’s identifier is truly unique (as this is needed to be able to match—sometimes more than one—pictures with one record) and being cognizant that some respondents may use more than one name and different spellings of the same name. It is critical that photos of vaccination records are of high quality so that dates are legible.
Equipment performance	Electronic tools should be adequately field tested prior to survey implementation. Field testing should be conducted early to avoid delays in survey implementation. Reopening data collection forms for completed surveys may introduce inaccuracies in the calculation of interview duration and related paradata.
Interviewer training and supervision	Quality training and supervision are very important. To ensure that data are of high quality, supervisors should provide adequate oversight.
Data analysis	Real-time analysis of field data is necessary for detecting potential challenges early.
**Example 5: Mali Survey Pilot (June–September 2022)**	
Interviewer training	Prior to interviewer training, it is useful to catalog all types of vaccination documentation interviewers are likely to encounter in the field and include photographs of each in the training materials. Interviewer training should be conducted well in advance of the main survey and be well organized. Ideally, trainees will visit enough households to ensure they encounter at least 5–10 vaccination cards that are challenging to decipher. Interviewers should be trained to expect that a significant percentage of caregivers will not have a HBR (necessitating reliance on caregiver recall) and many of the vaccination cards they encounter will be outdated and will thus not have spaces for all currently recommended vaccines.
Data entry quality control procedures	To ensure the data collected are of the highest quality possible, cross-checking the accuracy of data entered in the system against photographs of vaccination documentation is essential. Data entry should be cross-checked by multiple people (for example, both interviewers and supervisors) on an ongoing basis with a clear protocol for correcting data entry errors.
Data entry equipment	To facilitate quality control checks, it would be useful for the data collection program to flag potential errors and present vaccines in a table view that shows both vaccine names and dates. (e.g., if the date for Penta2 is before the date for Penta1, flag this record for review; code up a thorough list of expected relationships among the recorded dates, and flag records whose dates violate any of those logical expectations).
Household questionnaire	Questionnaires should be adapted locally–to include colloquial names for vaccines–as caregivers may not recognize standard vaccine names. Timeframe (e.g., referencing *the vaccination your child received at 9 months*), mode of administration (oral vs. injected), and color of vaccine vial can be effective cues for prompting caregiver recall of a child’s vaccination history.
**Example 6: Measles and Rubella Vaccination Campaign Evaluation Pilot Study, Burundi (2022)**	
Household enumeration process	To ensure sufficient time to enumerate all households and survey selected households, especially within semi-urban localities where the number of households is very high, increase the number of guides/community health workers assigned to each enumeration area.
Data collection protocol	Assign data collection teams responsible for collecting vaccination information in health facilities after interviewers provide them with the names of children who lack vaccination cards. When caregivers have been using a basic notebook to record their children’s vaccination information, data collectors should first transfer vaccination information from the notebook into an official vaccination card, photograph the completed card for the study records, and then provide the caregiver with the child’s completed vaccination card to retain for their records.
Household questionnaire	Add question to questionnaire to identify reason(s) a child did not participate in a vaccination campaign, including a response field to indicate when a child already received all doses of the vaccine provided as part of the campaign.
**Example 7: NSTOP Pilot, Nigeria (May 2014)**	
Training of supervisors and interviewers	Strengthen the supervisory structure because data collection staff have variable skill levels. Base final selection of interviewers on their performance during the training and post-training evaluation. If the project timeline is protracted, conduct refresher training of trainers prior to implementation of subsequent data collection phases, followed by a two-day cascade training for interviewers led by master trainers who participated in the previous phase.
Field navigation and cluster coverage	Due to security issues, command center staff were unable to travel outside Abuja but were able to use global positioning satellite (GPS) and satellite imagery to confirm household structures remotely. Survey teams found it helpful to use GPS navigation to locate their assigned clusters. Consequently, a full time GPS specialist was hired to be part of the command center team in Abuja for phases 1 and 2 of the main study. Due to long distances and difficult terrain between clusters, project leaders reduced expectations about the number of clusters that could be serviced by an interview team per day.

**Table 2:** Elements to pilot test: sampling procedures.

**First stage sampling—PSU frame and information assets**
Evaluate the sampling frame [12].
Evaluate the availability and quality of PSU maps and boundary geocoordinates.
Evaluate how long it will take to obtain the PSU maps from official government sources.
If applicable, evaluate other methods to select PSUs (e.g., gridded population sampling) [13].
**Second (and further) stage sampling**
Test the procedure for contacting community leaders to establish expectations, field questions, and clarify the dates of upcoming fieldwork.
Test household mapping/listing/sampling procedures [2,14] and modify, as needed, depending on the context (e.g., in cases where persons have been displaced due to natural disaster or insecurity).
Test the adequacy of the planned time to conduct mapping and listing within clusters.
Test the feasibility of sampling procedures of households and respondents within households [15,16,17]^.^
Estimate household listing response rate and evaluate methods to reduce nonresponse [12,15].
Test protocol concerning PSU segmentation, if applicable.
Assess interviewers’ ability to identify PSU boundaries.
Test the reliability of cluster/segment maps and feasibility of constructing new maps, if needed.
Test the protocols for definition of a household.
Test the protocols for inclusion of visitors, including potentially disadvantaged groups.
Test the protocols for other methods of sampling (e.g., use of satellite imagery) [18,19].
Test the procedures used to ensure the survey teams interview the households that have been selected, and that standard operating procedures are followed if household replacement is carried out.
Determine best days and time of day for conducting interviews in different settings (according to when residents able to provide vaccination information are most likely to be home). This may be particularly relevant where mothers often work outside the house.
When the protocol includes revisiting households where the interview is incomplete after the first visit, test the procedures for being able to navigate back to the correct household [20].
Test the procedures to re-open an interview record to resume a previously interrupted interview.

**Table 3:** Elements to pilot test: survey instruments.

**Test and refine the questionnaire**
Check understandability and the translation’s accuracy [11,21,22].
Assess the appropriateness of questionnaire length, keeping in mind respondent burden.
Assess the flow of questions, particularly the design of the section of the form to collect vaccination dates, in comparison to source documents (e.g., the order of vaccinations on the questionnaire should match the order on HBRs, if possible).
If the survey instrument is changed, re-test the flow of questions.
Assess what source documents are likely to be found in households and how much these vary (e.g., changes in HBR design over time, public versus private sector HBRs, HBRs from various countries) [23,24,25].
Ask participants for feedback to identify ambiguities and difficult questions. Discard or reword unnecessary, difficult or ambiguous questions [3,26].
Assess the adequacy of the method to assist respondents with identifying child’s age (e.g., calendar of local events) [27,28,29,30].
Assess the adequacy of the prompts/probes/diagram or printed memory aids to assist caregiver recall of the type of vaccine (e.g., vaccines usually administered on the arm vs. thighs), of place of vaccination and distinction between doses from routine immunization (RI) and from supplementary immunization activities (SIAs).
Conduct cognitive interviews to probe respondent thought processes and refine questions [31,32].
Refine answer options as needed [12,15].
Identify issues for which additional questions are needed [11].
Consider dropping questions with low response rates, or with little or no variation.
Shorten and revise the questionnaire and, if possible, pilot the questionnaire again.

**Table 4:** Elements to pilot test: field procedures.

**Test protocols for conducting household visits and interviews and HBR review**
Assess the adequacy of procedures for contacting respondents and/or following up with respondents [15] .
Assess the interviewers’ ability to establish rapport with respondents, obtain informed consent and ask questions in clear and unbiased way.
Assess the workflow with managing paper forms (interviewers and supervisors) [33].
Assess the workflow with computer-assisted personal interviewing (CAPI) devices [26]. This is particularly important if different target age groups are interviewed using different forms, for example about RI and SIAs.
Test procedures for entering/coding data [15] transmission, checking, and correction if a problem is identified.
Estimate interview response rate and evaluate methods to reduce nonresponse [3,12,15].
Visit some households and confirm that the styles of HBRs found there are included in the survey training materials. Test the protocol for photographing HBRs and linking multiple photographs with one record. If CAPI is used, test the reliability of phones or tablets, security of data, and ability to store and transmit data at different times of day/week.
**Test protocols for visiting health facilities to consult FBRs**
Test the protocols for pre-coordination (e.g., obtaining requisite permission/authorization, having registers available, having space to work).
Evaluate the availability, quality, and completeness of FBRs to decide if it’s worthwhile to include health facility visit.
Test the protocols for matching names, and other variables like family names, from household interview to register record.
Test the protocols for photographing FBRs and for assessing variability in photo quality.
Assess phone/tablet/camera memory card capacity for photos.
Test the protocol for backing up photos and other data collected at health facilities.
Test the protocols for data entry, transmission, checking, and correction if a problem is identified.
Assess the likelihood that FBR provides missing information or data that overturns caregiver recall.
Assess the protocol for merging FBR data with data from household interview.
Estimate average time per child (by age group) to find and record FBR data.
**Logistics and administration**
Assess the adequacy of the staff’s understanding after they complete training (e.g., what mistakes do they make or what misunderstandings persist?). Cycle back and improve the training materials. After training, assess the field staff’s understanding of staff safety protocols; revise the training as needed. Incorrectly coded ID variables (e.g., stratum ID, cluster ID, household ID, respondent ID, interviewer ID) are surprisingly common and time-consuming to correct. Evaluate procedures for minimizing those errors. Validate maps/geography/topography with trusted community leaders. Instruct data collection teams on navigational practices, such as a combination of using physical maps and GPS position overlaid on digital moving maps. Assess the efficiency of operations, and communication between office and field personnel.

**Table 5:** Elements to pilot test: data management, data quality, and data analysis.

**Data management**
Test procedures for data coding [15].
Test procedures for data cleaning [11].
Pretest process for handling images [34].
Test survey response database.
Test database(s) for information assets (lists of PSUs, PSU maps, driving directions, local contacts/drivers/officials associated with each PSU, lists of survey workers, system to account for labor days/hours, expenses, payroll, etc.). Stress-test the phones or tablets to ensure they have adequate memory and storage media to hold responses and photographs from a large number of interviews.
Test the capability of data collection reporting infrastructure (dashboards or reports) [35,36,37].
**Data quality**
For post-campaign coverage surveys (PCCS)/SIAs, assess the accuracy of background information gleaned about which vaccines were included in the SIA, and the site of injection. Determine whether finger marks were used and, if so, how to distinguish them if the SIA included multiple vaccinations; determine whether SIA-specific cards were given and whether the doses were written on the routine child health record.
Check that skip patterns are implemented correctly [15,26,38].
Estimate data entry error rates by interviewer, by team, and over time [39].
Assess the accuracy of recording responses on paper or digital devices by comparing data with photos.
Test the process for real-time identification and correction of omissions and data entry errors [40,41,42,43].
Assess the protocol for checking and correcting dates that trigger data quality flags. Ensure that data quality flags do not interrupt data entry. Avoid procedures that require solving a data entry error before continuing to enter the rest of the data in the questionnaire.
Check procedures for cleaning data [11], recoding, and creating new variables.
**Data analysis**
Check the procedures for data analysis. For example, use pilot data to test the database [44].
Estimate data processing time requirements [11].
If a field pilot yields a large sample of data, estimate intracluster correlation coefficient (ICC) and design effect and re-check the survey sample size calculation; adjust as appropriate [45,46,47,48,49,50].

## Dedication

We dedicate this paper to our late friend, Pierre Claquin, who passed away in 2021. Pierre designed and managed the Bangladesh pilot survey described here and was a coauthor of the revised World Health Organization cluster survey reference manual that the pilot informed. He was a gentleman and scholar, a compassionate physician, a proud smallpox warrior, and a talented photographer who appreciated people, fine food, wine, and conversation. We are grateful to have known him and labored alongside him.
